# Health Risks of Limited-Contact Water Recreation

**DOI:** 10.1289/ehp.1103934

**Published:** 2011-10-26

**Authors:** Samuel Dorevitch, Preethi Pratap, Meredith Wroblewski, Daniel O. Hryhorczuk, Hong Li, Li C. Liu, Peter A. Scheff

**Affiliations:** 1 Division of Environmental and Occupational Health Sciences, and; 2 Division of Epidemiology and Biostatistics, University of Illinois at Chicago School of Public Health, Chicago, Illinois, USA; 3 Institute for Environmental Science and Policy, University of Illinois at Chicago, Chicago, Illinois, USA

**Keywords:** environmental microbiology, epidemiology, gastrointestinal illness, wastewater, water pollution, water recreation

## Abstract

Background: Wastewater-impacted waters that do not support swimming are often used for boating, canoeing, fishing, kayaking, and rowing. Little is known about the health risks of these limited-contact water recreation activities.

Objectives: We evaluated the incidence of illness, severity of illness, associations between water exposure and illness, and risk of illness attributable to limited-contact water recreation on waters dominated by wastewater effluent and on waters approved for general use recreation (such as swimming).

Methods: The Chicago Health, Environmental Exposure, and Recreation Study was a prospective cohort study that evaluated five health outcomes among three groups of people: those who engaged in limited-contact water recreation on effluent-dominated waters, those who engaged in limited-contact recreation on general-use waters, and those who engaged in non–water recreation. Data analysis included survival analysis, logistic regression, and estimates of risk for counterfactual exposure scenarios using G-computation.

Results: Telephone follow-up data were available for 11,297 participants. With non–water recreation as the reference group, we found that limited-contact water recreation was associated with the development of acute gastrointestinal illness in the first 3 days after water recreation at both effluent-dominated waters [adjusted odds ratio (AOR) 1.46; 95% confidence interval (CI): 1.08, 1.96] and general-use waters (1.50; 95% CI: 1.09, 2.07). For every 1,000 recreators, 13.7 (95% CI: 3.1, 24.9) and 15.1 (95% CI: 2.6, 25.7) cases of gastrointestinal illness were attributable to limited-contact recreation at effluent-dominated waters and general-use waters, respectively. Eye symptoms were associated with use of effluent-dominated waters only (AOR 1.50; 95% CI: 1.10, 2.06). Among water recreators, our results indicate that illness was associated with the amount of water exposure.

Conclusions: Limited-contact recreation, both on effluent-dominated waters and on waters designated for general use, was associated with an elevated risk of gastrointestinal illness.

Limited-contact water recreation activities are popular in the United States. An estimated 71 million people participate in fishing, 52 million in motor boating, 20.7 million in canoeing, 9.4 million in rowing, and 6.4 million in kayaking (Cordell et al. 2004). Some waters that have not attained the goal of the Clean Water Act (1972) to support “recreation in and on the water” are used for limited-contact recreation (e.g., fishing and boating) but not full-contact recreation (e.g., swimming and water skiing). Recently, site-specific standards for limited (or secondary) contact recreation have been explored in several U.S. states for waters that do not support full-contact recreation, generally because of high concentrations of bacteria [Illinois Pollution Control Board 2010; Missouri Department of Natural Resources 2011; Texas Commission on Environmental Quality 2009; U.S. Environmental Protection Agency (EPA) 2003; Utah Department of Environmental Quality 2008]. Large cohort studies (Colford et al. 2007; Wade et al. 2008, 2010) have evaluated the health risks of full-contact recreation, but little is known about the health risks of limited-contact recreation. The Chicago Health, Environmental Exposure, and Recreation Study (CHEERS), a prospective cohort study, was designed to estimate the risk of illness attributable to limited-contact water recreation. Additionally, we assessed the severity of illness reported by study participants.

## Materials and Methods

*Overview.* The design and methods for the study presented here were adapted from those of the U.S. EPA’s National Epidemiological and Environmental Assessment of Recreational water (NEEAR) study (Wade et al. 2006, 2008, 2010). The CHEERS study, which was conducted by the University of Illinois at Chicago in the Chicago, Illinois, area between 2007 and 2009, included people who were engaged in limited-contact water recreation (defined as canoeing, fishing, kayaking, motor boating, or rowing) and people who were engaged in non–water recreational activities. After being screened for eligibility, participants underwent two field interviews: a brief prerecreation interview that collected contact information; an interview immediately after recreation inquired about demographics, dietary and other exposures, symptoms at baseline, and the extent of water exposure during recreation. Barcoded wrist bands were applied to the wrist or ankle of participants to ensure correct matching of data from pre- and postrecreation interviews from individual participants. On approximately days 2, 5, and 21, the participants were contacted by telephone and asked about exposures (including water recreation), the development of health symptoms, and the severity of symptoms, since the previous interview. Computer-assisted interviews were conducted in the field and by telephone using Blaise version 4.7 (Statistics Netherland; Heerlen, the Netherlands).

*Setting.* Participants were enrolled at Chicago-area locations where limited-contact water recreation takes place, including the Chicago Area Waterways System (CAWS). The CAWS, which includes the Chicago River, is engineered so that urban drainage and wastewater flow backward from Lake Michigan toward the Mississippi River. Two wastewater treatment plants that use an activated sludge process but no disinfection (such as chlorination) each discharge about 300 million gallons of treated wastewater per day into segments of the CAWS where limited-contact recreation (but not swimming) is permitted. Approximately 75% of the annual flow through the CAWS originates from the treatment plans (Rijal et al. 2011). In addition to the CAWS, recruitment took place at inland lakes, rivers, and Lake Michigan beaches designated by the state for swimming and other full-contact use, referred to here as general-use waters (GUW) [see Supplemental Material, Figure 1 (http://dx.doi.org/10.1289/ehp.1103934) for recruiting locations].

**Figure 1:**
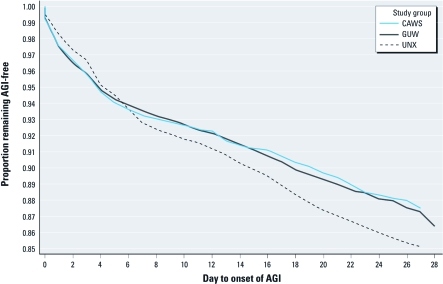
Kaplan–Meier curve of AGI survival, by study group.

We recently reported that in the 2009 participant recruiting season (April through July), geometric mean densities of *E. coli* and enterococci, which were analyzed by membrane filtration, were 678 and 164 colony-forming units (CFU)/100mL on the CAWS and 96 and 70 CFU/100 mL at the GUW locations, respectively (Dorevitch et al. 2011a). Detection of *Giardia* and adenovirus type F was more frequent on the CAWS (86% and 65% of samples, respectively) than on the GUW (47% and 24%, respectively) waters. *Cryptosporidium* and enterovirus were each detected in about 30% of samples at both CAWS and GUW locations (Aslan et al. 2011; Dorevitch et al. 2011a).

*Participants.* Limited-contact recreators were enrolled into one of two water recreation groups, CAWS or GUW, depending on their location of recreation. People who were engaged in non–water recreational activities at locations adjacent to the CAWS and GUW water access locations, including cycling, jogging, rollerblading, team sports, and walking, were enrolled into the unexposed (UNX) group. People were not eligible for enrollment in CHEERS if they had engaged in surface-water recreation (not including pools or water parks) within the previous 48 hr, intended to swim during their index recreation event, or would not be available for telephone follow-up. People were not excluded from the study because of unintentional swimming (e.g., falling into the water while kayaking). After completing the day-21 telephone follow-up interview, participants were allowed to re-enroll. Recruitment took place in 2007 (August through November), 2008 (March through October), and 2009 (April through July).

*Exposure assessment.* Self-reported exposure to recreational water was evaluated in the postrecreation interview. Participants who reported any water contact were asked to evaluate, by region of the body (i.e., head, face, torso, upper extremity, and lower extremity), their degree of water exposure. Exposure was scored as none (0), drops (1), splash (2), drenched (3), or submerged (4). Scores were summed by body region (values ranged from 0 to 4), and overall (scores ranged from 0 to 16). Water ingestion was categorized as none, drops, teaspoon, mouthful. The validation of the self-reported estimates of water ingestion has been reported previously (Dorevitch et al. 2011b).

*Definitions of the five health outcomes.* Acute gastrointestinal illness (AGI) was defined as *a*) three episodes of diarrhea in 24 hr, *b*) vomiting, *c*) nausea with stomachache, *d*) nausea that interferes with daily activities, or *e*) stomachache that interferes with daily activities; this definition for AGI was used by the NEEAR study (Wade et al. 2006). Acute respiratory illness (ARI) was defined as *a*) fever plus nasal congestion, *b*) fever plus sore throat, or *c*) cough with phlegm; this definition for ARI was used in the Mission Bay study on swimming (Colford et al. 2007). Participants were classified as having ear symptoms if they reported any ear pain or ear infection; as having eye symptoms if they reported any eye redness, crusting, itching, or draining; and as having a skin rash if they reported any rash. An individual who had baseline symptoms of a particular outcome was not considered to be at risk for that outcome but was at risk for developing other health outcomes.

*Data analysis.* We identified potential effect modifiers and confounders of associations between the exposure groups and outcomes using conceptual models based on prior studies and principles of infectious disease epidemiology [see Supplemental Material, Table 1 (http://dx.doi.org/10.1289/ehp.1103934), for a complete list of the variables identified]. We determined time windows of interest for each health outcome using survival analysis methods, beginning with the generation of Kaplan–Meier plots. If log-negative–log survival plots did not identify time windows of differential risk across study groups (i.e., time-by-group interactions), we determined time windows of interest based on incubation periods described in the literature for pathogens previously identified as causes of outbreaks of recreational waterborne illness (Dziuban et al. 2006; Yoder et al. 2008). However, we also repeated analyses using several different time windows for illness onset to evaluate the sensitivity of effect estimates to the definition of time window for each outcome.

**Table 1:** Demographic and water recreational activities of CHEERS participants [*n* (%)] by study group.

Variable	Category	CAWS *n *= 3,966	GUW *n* = 3,744	UNX *n* = 3,587
Race/ethnicity		White		3,047 (76.9)		3,077 (82.2)		2,274 (63.5)
		Black/African American		286 (7.2)		126 (3.4)		574 (16.0)
		Hispanic		208 (5.2)		246 (6.6)		340 (9.5)
		Other/multiple		422 (10.7)		291 (7.8)		392 (11)
		Missing		3		4		7
Age (years)		0–4		33 (0.8)		37 (1.0)		62 (1.7)
		5–9		147 (3.7)		182 (4.8)		110 (3.1)
		10–17		403 (10.1)		369 (9.9)		193 (5.4)
		18–44		2,328 (58.7)		1,730 (46.2)		1,830 (51.0)
		45–64		924 (23.3)		1,279 (34.2)		1,175 (32.8)
		≥ 65		131 (3.3)		147 (3.9)		217 (6.0)
Sex		Female		1,982 (50.0)		1,512 (40.4)		1,829 (51.0)
		Male		1,984 (50.0)		2,232 (59.6)		1,758 (49.0)
Water recreation activity		Motor boating		661 (16.7)		232 (6.2)		
		Canoeing		885 (22.3)		1,202 (32.1)		
		Fishing		425 (10.7)		858 (22.9)		
		Kayaking		1,355 (34.2)		1,200 (32.1)		
		Rowing		640 (16.1)		252 (6.7)		
Face/head wetness		Not wet		2,003 (50.5)		2,506 (66.9)		
		Sprinkle/drops		1,366 (34.4)		721 (19.3)		
		Splash		554 (14.0)		376 (10.0)		
		Drenched		28 (0.7)		33 (0.9)		
		Submerged		15 (0.4)		108 (2.9)		
Swallowed water		None		3,794 (95.7)		3,614 (96.5)		
		Drops		120 (3.0)		78 (2.1)		
		Teaspoon		43 (1.1)		38 (1.0)		
		Mouthful		9 (0.2)		14 (0.4)		
Other than race/ethnicity information, no data were missing.								

We estimated the AORs for each outcome using logistic regression models that included all covariates identified in the conceptual models as potential confounders, as well as all potential effects modifiers (interaction terms) that were found to be significant at *p* < 0.10. In the absence of multicolinearity, covariates were not eliminated from the models. Models of AGI and ARI included terms for water ingestion (dichotomized as a mouthful or more vs. less), while models of ear and eye symptoms included a term of face wetness (the eye model also included a term for hand wetness, because we considered hand-to-eye contact to be a route of exposure), and skin rash included the total dermal exposure score. Associations between water recreation and illness were evaluated in three-group models using two indicator variables (CAWS vs. other, GUW vs. other, with UNX as the reference category). Differences in illness attributable to wastewater (as opposed to other surface water) exposure were evaluated using data from CAWS and GUW groups only; we adjusted these models for self-reported water exposure and specific water recreation activities.

Attributable risk was defined as the incidence proportion in the exposed group minus the incidence proportion in the UNX group, which was adjusted for differences in the distribution of covariates between groups. These estimates were computed using a counterfactual exposure scenario, which implemented the G-computation method as described by Fleischer et al. (2010). For each health outcome, coefficients of the logistic model were used to calculate the predicted probability of illness of each individual, using his or her unique values for all covariates except for the study group. Instead of the observed group of the participant, the counterfactual for CAWS forced every participant’s value for group to be CAWS, regardless of the group in which the participant had been enrolled in the field study. Similarly, the counterfactual for GUW and UNX forced every participant’s value for group to be GUW and UNX, respectively. These predicted probabilities of illness were averaged by group to produce a counterfactual point estimate of risk for CAWS, GUW, and UNX. Attributable risk differences were computed by subtracting the average counterfactual probability of illness of one group from a comparison group. A bias-corrected 95% confidence interval (CI) was obtained using the standard CI (Efron and Tibshirani 1986). Using the SURVEYSELECT procedure in SAS (version 9.2; SAS Institute Inc., Cary, NC), we sampled with replacement from the study sample of 11,297 observations to obtain 1,000 bootstrap samples of the same size as the original. For each of these samples, the multivariate logistic models were fit, and the G-computation method was used to calculate the risk differences between study groups. The distribution of 1,000 bootstrap risk differences was used to estimate standard 95% CIs around the point estimates of attributable risk.

Additional analyses included the use of random effects models (PROC GLIMMIX; SAS) to evaluate whether data obtained from individuals who participated more than once could be considered independent of data collected from those same individuals during prior participation in the study. We did not perform random-effects analyses of family units because < 5% of participants enrolled with family members. Propensity scores were used in the logistic models to evaluate potential confounding that may have resulted from the absence of randomization of participants to study groups. Propensity score methods were employed to match participants from different groups according to their observed covariate values (Rosenbaum and Rubin 1983). Two propensity scores, the probabilities of being in CAWS versus UNX and GUW versus UNX, were calculated for each participant using a logistic model predicting the individual’s group (CAWS vs. UNX and GUW vs. UNX) based on the observed covariate values for the individual. Participants were then categorized into strata according to their propensity scores, resulting in covariate-matched individuals in each stratum. Stratified group effects were compared with the multivariate logistic result that did not include propensity score strata. All data analyses were performed using SAS, version 9.2.

*Human subjects research.* Participants (or a parent or guardian if < 18 years of age) provided written documentation of informed consent following a protocol approved by the Institutional Review Board of the University of Illinois at Chicago.

## Results

*Participants.* A total of 11,733 sets of field interviews were completed, of which 11,297 (96.3%) were associated with telephone follow-up [for details about attrition, see Supplemental Material, Figure 2 (http://dx.doi.org/10.1289/ehp.1103934)]. Of the 11,297 sets of field and telephone interviews, 10,646 (94.2%) were obtained from individuals who participated one time, 4.6% were obtained from individuals who participated twice, and 1.1% were obtained from individuals who participated more than twice. Participants were enrolled on 390 location dates over 190 dates. The number of participants and the distribution of their ages were similar across the three groups (Table 1). Among the two water recreation groups, rowing and motor boating were more common among CAWS participants, whereas fishing and canoeing were more common among GUW participants.

*Time periods of interest.* In the first days after participation in the field study, the two water recreation groups had a higher proportion of incidence of AGI than did the UNX group (Figure 1), whereas starting at about day 7, AGI occurred more frequently among the UNX. Based on the log-negative–log survival curves, time-by-group interactions were present for AGI when dividing the follow-up period, for example, for days 0–2 versus days 3–21 and days 0–3 versus days 4–21 (i.e., the proportional hazards assumption was not valid). Curves for other end points did not suggest specific time windows for exposure outcome relations [see Supplemental Material, Figure 3A–3D (http://dx.doi.org/10.1289/ehp.1103934)]. Therefore, time windows were chosen based on reported incubation periods for pathogens of concern. The time period of interest for ARI was days 0–7, based on the incubation period observed in recreational waterborne *Legionella* outbreaks (Campese et al. 2010; Foster et al. 2006; Greig et al. 2004). For otitis externa, we used days 0–7, consistent with a prior epidemiologic study (Springer and Shapiro 1985). A 0- to 3-day time window of interest for skin rash was based on prior studies of outbreaks of cercarial dermatitis, which generally occurs within the first 2 days of water recreation [Centers for Disease Control and Prevention (CDC) 1992; Hoeffler 1977; Mulvihill and Burnett 1990].

*Incidence of illness and associations between illness and group.* The incidence proportion of each health outcome is summarized by group in Table 2, indicating that gastrointestinal, eye, and skin symptoms were more frequent than respiratory or ear symptoms. The AOR and CIs for associations between study group and illness are summarized in Table 2, and complete model estimates (including AORs for model covariates) are provided in Supplemental Material, Tables 2A–2E (http://dx.doi.org/10.1289/ehp.1103934). Study group was associated with AGI incidence. For the CAWS versus UNX comparison, the AOR was 1.46 (1.08, 1.98) and for the GUW versus UNX comparison, 1.50 (1.09, 2.07). No association was suggested between AGI and whether limited-contact recreation took place on effluent-dominated (CAWS) versus other (GUW) waters. The adjusted odds of eye symptoms among CAWS participants were elevated, with either UNX or GUW as the reference group.

**Table 2:** Incidence proportion by study group and AORs for associations between study group and health outcome.

Incidence (per 100)	Three-group model		CAWS vs. GUW	
	Outcome	Group	Cases/total	AOR (95% CI)		AOR (95% CI)	
AGI		CAWS		163/3,793		4.30		1.46	(1.08, 1.96)		1.02	(0.80, 1.31)
		GUW		152/3,575		4.25		1.50	(1.09, 2.07)		Ref	
		UNX		116/3,379		3.43		Ref				
ARI		CAWS		60/3,236		1.85		0.90	(0.57, 1.42)		0.94	(0.64, 1.38)
		GUW		70/3,089		2.27		1.04	(0.65, 1.67)		Ref	
		UNX		59/2,795		2.11		Ref				
Ear		CAWS		48/3,786		1.27		1.20	(0.70, 2.07)		1.03	(0.65, 1.63)
		GUW		41/3,560		1.15		1.13	(0.63, 2.01)		Ref	
		UNX		36/3,387		1.06		Ref				
Eye		CAWS		162/3,745		4.33		1.50	(1.10, 2.06)		1.34	(1.02, 1.77)
		GUW		113/3,501		3.23		1.17	(0.83, 1.65)		Ref	
		UNX		108/3,327		3.25		Ref				
Skin		CAWS		163/3,891		4.19		0.86	(0.64, 1.15)		1.18	(0.91, 1.54)
		GUW		133/3,655		3.64		0.72	(0.52, 1.00)		Ref	
		UNX		150/3,490		4.30		Ref				
Ref, reference. Covariates included in the models are listed in Supplemental Material, Tables 2A–2E (http://dx.doi.org/10.1289/ehp.1103934). The number of participants at risk for each outcome varied based on the number with symptoms for each outcome at baseline. For AGI, rash, and eye symptoms, the time window of interest was days 0–3. For respiratory and ear symptoms, the window was days 0–7.												

Two potential modifiers of associations between group and outcome were significant at the *p* = 0.1 level. An interaction between age category and study group for the incidence of ARI was suggested (*p* = 0.08). Among those ≤ 10 years of age, the AOR for developing ARI for CAWS versus UNX was 1.90 (0.67, 21.56), whereas for those ≥ 11 years of age, the AOR was 0.89 (0.56, 1.42). An interaction between diabetes and study group and the incidence proportion of AGI was observed (*p* = 0.04); the AOR for developing AGI in the CAWS versus UNX group was 1.52 (1.12, 2.07) for those without diabetes and 0.62 (0.19, 2.02) for those with diabetes. However, given the unstable estimates for the two smaller groups—those < 10 years of age with ARI (*n* = 13 in the CAWS + UNX groups) and those with diabetes (*n* = 14 with AGI in CAWS + UNX groups)—these health outcomes were modeled without interaction terms to arrive at more interpretable results.

Among those who participated in limited-contact recreation, swallowing water was associated with the occurrence of AGI and ARI, face wetness score was associated with ear symptoms, and hand wetness score was associated with eye symptoms (Table 3).

**Table 3:** Associations between exposure metrics and health outcomes in final multivariate models.

Outcome	Exposure metric	Exposed	*n* with outcome	*n* without outcome	AOR (95% CI)		*p*-Value for exposure term
AGI		Swallow mouthful		Yes		5		18		5.74	(2.05, 16.04)		< 0.001
				No		303		6,819					
ARI		Swallow mouthful		Yes		3		14		10.89	(2.95, 40.20)		< 0.001
				No		123		5,975					
Ear		Face wet score		—*a*		88		7,031		1.48	(1.20, 1.84)		0.007
Eye*b*		Face wet score		—*a*		270		6,751		1.12	(0.97, 1.29)		0.13
		Hand wet score		—*a*						1.21	(1.09, 1.35)		< 0.001
Skin		Total wet score		—*c*		288		7,016		1.02	(0.98, 1.06)		0.26
Numbers of participants are slightly different than those shown in Table 2. Data presented here are from multivariate models, which had some missing covariate data. **a**This exposure metric has five levels. Illness by exposure level data is not presented. **b**Wet score and face wet score were in the same model of eye symptoms. **c**This exposure metric has 16 levels. Illness by exposure level data is not presented.													

Risk difference (RD) estimates based on G-computation indicated greater risks for both water recreation groups compared with the UNX groups. For the CAWS and GUW groups, the RD (95% CI) relative to the UNX group were 13.7 (3.1, 24.9) and 15.1 (3.1, 24.9), respectively, per 1,000 recreators. No suggestion of RD for AGI was apparent between two water recreation groups (0.8; –9.9, 10.4) (Table 4). Consistent with logistic regression estimates, the RD for eye symptoms was significantly elevated for CAWS versus UNX (RD 14.3; 2.3, 24.4) but not for GUW versus UNX.

**Table 4:** Estimated cases of illness attributable to limited-contact water recreation per 1,000 recreators, by outcome and by group contrast.

CAWS vs. UNX		GUW vs. UNX		CAWS vs. GUW	
Outcome	RD (95% CI)		RD (95% CI)		RD (95% CI)	
AGI		13.7	(3.1, 24.9)		15.1	(2.6, 25.7)		0.8	(–9.9, 10.4)
ARI		–2.2	(–11.9, 6.9)		0.9	(–9.9, 11.2)		–1.4	(–9.9, 6.3)
Ear		2.1	(–5.7, 7.8)		1.3	(–5.4, 7.1)		0.4	(–4.4, 5.1)
Eye		14.3	(2.3, 24.4)		5.0	(–6.0, 14.3)		10.5	(–0.1, 20.3)
Skin		–6.3	(–18.9, 6.5)		–12.6	(–26.7, 1.1)		4.6	(–2.7, 14.6)
Estimates based on G-computation using all terms in the multivariate logistic models.									

*Evaluation of data analysis assumptions.* The final logistic model for AGI was re-run on data sets that defined AGI as the occurrence of symptoms during different time windows. AORs for association between AGI and CAWS group (with UNX as the reference) with 0–2, 0–4, and 0–5 days after exposure were 1.45 (1.04, 2.02), 1.23 (0.95, 1.60), and 1.18 (0.92, 1.51), respectively, whereas the estimate for the default (0- to 3-day) time window was 1.46 (1.09, 1.96). Similar findings (higher AORs for AGI with shorter time intervals) were noted for the GUW group and also for the association between CAWS recreation and eye symptoms (data not presented). The addition of terms for propensity score strata to the multivariate logistic models resulted in minimal changes (approximately 1%) in AORs, suggesting that despite the nonrandom design, confounding was not apparent after adjustment for model covariates. The GLIMMIX model of AGI demonstrated that a random-effect term for individual participants was not significant (likelihood ratio *p* = 0.13), consistent with the assumption that repeated participation events by individuals who enrolled more than once could be considered independent. The impact of the definition of exposure (swallowing water vs. head/face wetness) and degree of exposure (none vs. other, none or drop vs. other, etc.) on associations between study group and AGI were evaluated. Regardless of the definition of exposure, the AOR for group (CAWS vs. GUW) was approximately 1.0, confirming no difference between the adjusted odds of AGI for CAWS and GUW groups, as summarized in Supplemental Material, Table 3 (http://dx.doi.org/10.1289/ehp.1103934). Last, we attempted to run binomial models of health outcomes as was peformed by Wade et al. (2008) in their analyses of the NEEAR study; however, models did not converge, as may be expected for uncommon (< 10% incidence) events (McNutt et al. 2003; Zou 2004).

*Symptom severity.* Of the 431 participants who developed AGI (including those who developed other symptoms along with AGI), there were no significant differences (*p* > 0.05) among exposure groups in the proportion who took over-the-counter medication or saw or spoke with a health care provider (45.2% and 12.5% for all participants combined). However, participants in the UNX group with AGI were more likely to report prescription medication use (9.4% compared with 4.3% and 4.4% in the CAWS and GUW groups; Fisher’s exact *p* = 0.002) and emergency department or hospital care [4.3% compared with 0% and 1% for CAWS and GUW (based on six cases of emergency department/hospital care), Fisher’s exact *p* = 0.008]. Among the 383 participants who developed eye symptoms, differences in severity by group were not apparent. Overall, 50.9% used over-the-counter medication, 5.7% used prescription medication, and 14.1% spoke with or saw a health care provider.

## Discussion

We observed risks of gastrointestinal illness attributable to limited-contact water recreation that were comparable whether the recreation took place on effluent-dominated waters or GUW. A risk of eye symptoms after limited-contact water recreation on effluent-dominated waters only was apparent. Two cohort studies, both set on the same United Kingdom whitewater canoeing slalom course fed by wastewater, reported associations between canoeing and the development of gastrointestinal illness (Fewtrell et al. 1992; Lee et al. 1997). However, substantial water contact, including capsize, occurred frequently (Lee et al. 1997), blurring the distinction between limited- and full-contact recreation. The risk of AGI after swimming at Great Lakes (Wade et al. 2006, 2008) and marine beaches (Wade et al. 2010) impacted by wastewater discharge, a marine beach not impacted by wastewater discharge (Colford et al. 2007), and an inland reservoir that does not directly receive wastewater discharge (although some of its tributaries do) have been described recently (Marion et al. 2010). Table 5 summarizes the unadjusted incidence proportion of illness and associations between exposure group and gastrointestinal illness in these studies and CHEERS. Caution should be used in comparing estimates of association and risk across studies because of differences in protocols (we interviewed individuals rather than family units), the frequency and timing of health follow-up (days 2, 5, and 21 in CHEERS vs. a single follow-up interview between days 8 and 14, depending on the study), and differences in the definition of gastrointestinal illness. We identified a relatively brief time window during which differences among groups in the incidence proportion of AGI was maximized. It is possible that stronger associations between illness and swimming might have been obtained in the earlier studies had the day 0–3 time windows been used for defining gastrointestinal illness.

**Table 5:** Comparison of findings of recent full-contact and limited-contact observational studies that included an unexposed group.

Definition of GI illness	Definition of water recreation	Unadjusted AGI cases/1,000	AOR (95% CI) for GI illness
Marine beach impacted by wastewater (Wade et al. 2010 )						
Any vomiting OR three loose stools/24 hr OR nausea with stomachache or that interfered with activity OR stomachache that interfered with activity		Swimmer: immersion to waist or higher		Swimmers, days with EN > 35 CFU: 108		Swimmer, EN > 35 CFU vs. nonswimmer: 1.52 (0.96, 2.4)
				Swimmers, days with EN < 35 CFU: 77		
				Nonswimmer: 59		
				Difference (> 35 vs. nonswimmer): 49		
Marine beach not impacted by wastewater discharge (Colford et al. 2007)						
Vomiting OR diarrhea with fever OR cramps and fever		Any water contact		Any water contact: 29		Any water contact vs. none: 0.96 (0.68, 1.4)
				Nonswimmer: 23		
				Difference: 6		
Freshwater beaches impacted by wastewater discharge (Wade et al. 2008)						
Same as Wade et al. 2010		Swimmer: immersion to waist or higher		Swimmer: 83		Swimmer vs. nonswimmer: 1.44 (1.27, 1.64)
				Nonswimmer: 60		
				Difference: 23		
Freshwater reservoir no immediate wastewater discharge (Marion et al. 2010)						
GI illness: nausea OR stomachache, OR diarrhea OR vomiting		Swimmer: wade, swim, or play in the water		Swimmer: 56		Swimmer vs. nonswimmer: 3.2 (1.1, 9.0)
				Nonswimmer: 19		
				Difference: 37		
Inland flowing and impounded waters (present study)						
See Wade et al. 2010		Limited-contact activity (boating, canoeing, fishing, boating, or rowing)		Limited contact: 43		CAWS vs. UNX: 1.46 (1.08, 1.96)
				UNX: 34		GUW vs. UNX: 1.50 (1.08, 1.96)
				Difference: 9		
Abbreviations: EN, enterococci; GI, gastrointestinal. Some of the studies listed used definitions of exposure and gastrointestinal illness other than those listed above.						

Given the above caveats, the risk of AGI attributable to limited-contact recreation appears to be within the range of attributable risk suggested by the above-noted studies of swimming. The incidence of AGI attributable to limited-contact water recreation was similar (about 14–15 cases/1,000) for the CAWS and GUW and groups, which is counterintuitive given that the CAWS is predominantly wastewater and that *Cryptosporidium* and adenovirus type F were more likely to be detected on CAWS compared with GUW locations (Aslan et al. 2011; Dorevitch et al. 2011a). This finding has potential policy implications, as the incidence proportion among GUW recreators is greater than the 8/1,000 targeted risk level at Great Lakes beaches established by the BEACH Act of 2004 amendments to the Clean Water Act (U.S. EPA 2004). Although CAWS and GUW recreators were equally likely to swallow a mouthful of water, GUW recreators reported head/face submersion more frequently than did CAWS recreators (2.9% vs. 0.4%; *p* < 0.001). Thus, the average dose of ingested pathogens (pathogen density per unit volume of water × volume of water ingested) may have been comparable for the two groups, with CAWS recreators experiencing head immersion less frequently, but in waters with higher pathogen densities, whereas GUW recreators experienced head immersion more frequently, but in waters with lower pathogen densities.

The higher incidence proportion of eye symptoms among CAWS recreators, compared with either users of GUW or the non–water recreators, stands in contrast to other cohort studies of comparable or larger size that did not identify statistically significant associations between swimming and eye symptoms (Colford et al. 2007; Wade et al. 2008). Canoeing on a whitewater slalom course in the United Kingdom was not associated with eye symptoms (Lee et al. 1997), although that study had less statistical power because of a sample size that was about one-tenth of CHEERS. U.S. recreational waterborne disease outbreaks have identified cases of eye symptoms (Dziuban et al. 2006; Yoder et al. 2008); however, these outbreaks occurred at spas and water parks that use disinfectants and other eye irritants. In addition to the possibility that infectious agents in the CAWS were responsible for symptoms, another possibility is that endotoxin, a component of the cell walls of gram-negative bacteria, played a role. Endotoxin has been thought to cause a variety of symptoms, including eye symptoms, among workers in wastewater treatment plants (Lee et al. 2007). Airborne gram-negative bacteria have been measured in the vicinity of one of the CAWS wastewater treatment plants (Scheff et al. 1981), although not specifically along the waterway.

Ambient water quality criteria have been established based on an estimated number of cases of illness attributable to swimming per 1,000 uses, rather than measures of association (such as odds ratios or relative risks) reported by epidemiologic studies. To estimate illness incidence as a function of water quality, the U.S. EPA criteria documents for fresh (U.S. EPA 1984) and marine (U.S. EPA 1983) beaches modeled the difference in AGI between swimmers and nonswimmers at beaches based on data collected over a season at a beach, and more recently, based on daily measures of indicators (Wade et al. 2008). We applied a recently described causal inference approach (Fleischer et al. 2010). This method, which makes use of the complete data set, may be useful in other observational studies in which estimates of attributable risk (defined as adjusted between-group differences in incidence) are desirable.

Strengths of this research include the relatively large sample size, prospective collection of exposure data, limited loss to follow-up, use of the same questionnaire items as previous studies, the data-driven approach to determination of time windows of concern for AGI, and the evaluation of illness severity. Confounding would have been reduced had participants been randomized to study groups, as has been done in controlled exposure studies (Fleisher et al. 2010) This approach was considered but not used, because participants in organized canoeing, kayaking, and rowing events would not have accepted randomization to non–water recreation. The unique aspects of the wastewater management system in Chicago, which unlike most U.S. cities does not include disinfection, may limit generalizability to other settings. This publication does not include evaluations of microbial measures of water or estimated dose (volume of water ingested × microbe density) as predictors of illness; these will be subjects of future manuscripts.

## Conclusions

It is generally assumed that risks of adverse health outcomes due to limited-contact water recreational activities such as boating, canoeing, fishing, kayaking, and rowing are relatively low, even on waters with high densities of microbial pollutants. We observed an increased risk of AGI associated with limited-contact recreation, both on effluent-dominated waters and on surface waters designated for full-contact use. The absence of a difference in risk of AGI between exposure to effluent-dominated waters and other waters was contrary to expectations given differences in the levels of potential pathogens. It is possible that differences in pathogen concentrations may have been offset by higher levels of water ingestion during recreation on water approved for general use. The risk of eye symptoms was elevated among those who engaged in limited-contact recreation on effluent-dominated waters only, possibly because of infectious agents, endotoxin, and/or irritants. Ear, skin, and respiratory symptoms were not associated with limited-contact recreation. The occurrence of gastrointestinal, respiratory, eye, and ear symptoms was strongly associated with the degree of self-reported water exposure, suggesting that observed associations between water recreation and illness are causal.

## Supplemental Material

(410 KB) PDF
