# Supplementary material for: Health Risks of Limited-Contact Water Recreation
Source: Environ Health Perspect. 2011 Oct 26;120(2):192–7. doi: 10.1289/ehp.1103934 (PMC3279449; doi:10.1289/ehp.1103934)

SUPPLEMENTARY MATERIAL

Health Risks of Limited-contact Water Recreation

Samuel Dorevitch, Preethi Pratap,, Meredith Wroblewski, Daniel O. Hryhorczuk, Hong Li, Li C. Liu, Peter A. Scheff

List of supplementary material

1. Supplementary Material, Table 1: Potential confounders and effect modifiers
2. Supplementary Material, Table 2a: results of multivariate logistic regression model of AGI
3. Supplementary Material, Table 2b: results of multivariate logistic regression model of ARI
4. Supplementary Material, Table 2c: results of multivariate logistic regression model of ear symptoms
5. Supplementary Material, Table 2d: results of multivariate logistic regression model of eye symptoms
6. Supplementary Material, Table 2e: results of multivariate logistic regression model of skin rash
7. Supplementary Material, Table 3: alternative definitions of exposure in analyses of 2-group models of AGI
8. Supplementary Material, Figure 1: CHEERS recruiting locations (North: Figure 1a, and South, Figure 1b)
9. Supplementary Material, Figure 2: Participant Enrolment and Attrition
10. Supplementary material, Figure 3a: Survival curve, acute respiratory illness (ARI)
11. Supplementary material, Figure 3b: Survival curve, ear symptoms
12. Supplementary material, Figure 3c: Survival curve, eye symptoms
13. Supplementary material, Figure 3d: Survival curve, skin rash

<u>Supplementary Material Table 1: Potential confounders and effect modifiers</u>

**Potential confounders and effect modifiers of associations between study group and outcome**	**AGI**	**ARI**	**Ear**	**Eye**	**Skin**
Age category	C, E	C, E	C, E	C, E	C, E
Gender	C	C	C	C	C
Race/ethnicity	C	C	C	C	C
Recent contact with dog, cat	C	C			C
Recent contact with other animals	C	C			
Recently ate shell fish, sushi	C				C
Recently ate undercooked meat	C				
Recently ate raw/runny eggs	C				
Recently ate packaged sandwich	C				
Recently ate hamburger	C				
Chronic GI condition	C, E	C			
Baseline average number of bowel movements/day	C, E				
Chronic respiratory condition		C, E	C		
Recent contact with someone who has GI symptoms	C		C	C	
Recent contact with someone who has respiratory symptoms		C		C	
Recent contact with someone who has eye symptoms				C	
Diabetes	C, E	C, E	C, E	C, E	C, E
Recent antibiotic use	C	C			C
Recent antacid use	C	C			
Prone to infection	C, E	C, E	C, E	C, E	C, E
Pre-existing bug bites, cuts or sunburn					C
Eating, hand washing during recreation	C	C			
Water recreation during follow-up	C	C	C	C	C
Perceived risk of CAWS recreation	C, E	C, E	C, E	C, E	C
Frequency of recreation at location of enrollment	C	C	C	C	C

C: Potential confounder, E: Potential effect modifier

Supplementary Material Table 2a: results of multivariate logistic regression model of AGI

Predictor	Ref./scale	All three groups		Water recreators only	
		OR	95% CI	OR	95% CI
CAWS group	UNX	1.46*	1.08, 1.96		
GUW group	UNX	1.50*	1.09, 2.07		
CAWS group	GUW			1.02	0.80, 1.31
Age ≤10 yr	11-64 yrs	0.54*	0.32, 0.90	0.42*	0.22, 0.80
Age ≥65 yrs	11-64 yrs	0.33*	0.15, 0.70	0.40*	0.17, 0.92
Male	Female	0.77*	0.63, 0.95	0.75*	0.59, 0.95
White	African Amer.	0.50**	0.36, 0.68	0.55*	0.35, 0.87
Hispanic	African Amer.	0.72	0.47, 1.10	0.72	0.40, 1.29
Other	African Amer.	0.62*	0.41, 0.94	0.69	0.39, 1.20
Same water use: 5-10 days	0-4 days	1.46*	1.10, 1.95	1.34	0.95, 1.90
Same water use: >10 days	0-4 days	0.86	0.61, 1.20	0.77	0.50, 1.19
Contact with person w/ GI ‡‡	None	1.32	0.84, 2.06	1.41	0.82, 2.44
Chronic GI condition	None	2.11**	1.44, 3.08	2.43**	1.57, 3.74
Perceived risk of CAWS recreation	0-10 scale	1.08**	1.04, 1.12	1.08*	1.03, 1.12
Baseline avg. # of daily bowel movements	0-3	1.29*	1.11, 1.49	1.23*	1.03, 1.46
Dog or cat contact‡	None	0.97	0.78, 1.19	0.89	0.70, 1.14
Other animal contact‡	None	1.22	0.86, 1.75	0.96	0.62, 1.48
Are raw/undercooked eggs‡	None	1.18	0.75, 1.87	1.05	0.59, 1.88
Ate raw/undercooked meat‡	None	1.07	0.67, 1.73	1.41	0.84, 2.38
Ate hamburger‡	None	1.23	0.99, 1.53	1.19	0.92, 1.54
Ate fresh fruit/vegetable‡	None	0.90	0.65, 1.23	0.80	0.56, 1.15
Ate shellfish, sushi‡	None	1.08	0.72, 1.60	1.03	0.63, 1.69
Ate pre-packaged sandwich‡	None	1.41	0.97, 2.06	1.26	0.80, 1.98
Has diabetes	Does not	1.46	0.87, 2.45	0.83	0.40, 1.75
Took antibiotics in past 7 days	No	1.18	0.74, 1.86	0.88	0.47, 1.64
Is prone to infection	Not	0.84	0.45, 1.57	0.66	0.28, 1.54
Anta95% CId use in past 48 hours	None	1.30	0.92, 1.83	1.24	0.83, 1.84
Water recreation, follow-up days 0-3	None	1.12	0.87, 1.44	0.95	0.69, 1.30
Ate/drank , washed hands	Didn't eat/drink	0.92	0.66, 1.28	0.85	0.60, 1.19
Ate/drank, did not wash hands	Didn't eat/drink	0.94	0.72, 1.23	0.95	0.72, 1.25
Canoeing	Motor boating		N/A	0.74	0.50, 1.10
Kayaking/rafting	Motor boating		N/A	0.73	0.50, 1.07
Rowing	Motor boating		N/A	0.47*	0.27, 0.82
Fishing	Motor boating		N/A	1.04	0.67, 1.61
Swallowed ≥mouthful of water	Did not		N/A	5.74*	2.05, 16.04

Acute gastrointestinal Illness (AGI) *2-sided P value <0.05; **<0.001

‡ ‡ Past 72 hours

Supplementary Material, Table 2b: results of multivariate logistic regression model of ARI

Predictor	Ref./scale	All three groups		Water recreation groups	
		OR	95% CI	OR	95% CI
CAWS group	UNX group	0.90	0.57, 1.42		
GUW group	UNX group	1.04	0.65, 1.67		
CAWS group	GUW group			0.94	0.64, 1.38
Age <11 yr	Age >10	1.01	0.55, 1.85	1.14	0.59, 2.22
Male	Female	1.13	0.84, 1.52	1.27	0.87, 1.83
White	African Amer.	0.76	0.44, 1.30	0.75	0.35, 1.62
Hispanic	African Amer.	1.60	0.84, 3.07	1.10	0.44, 2.79
Other	African Amer.	1.03	0.53, 2.00	1.16	0.48, 2.83
Same water use: 5-10 days	0-4 days	1.18	0.75, 1.87	0.94	0.52, 1.70
Same water use: >10 days	0-4 days	0.99	0.61, 1.61	0.75	0.38, 1.47
Perceived risk of CAWS recreation	0-10 scale	1.01	0.95, 1.06	1.01	0.95, 1.08
Exposure in past 48 hr					
Dog or cat	None	1.45*	1.05, 2.01	1.71*	1.13, 2.59
Other animal contact	None	1.27	0.75, 2.14	1.16	0.62, 2.15
Exposure to someone with GI illness‡‡	None	1.48	0.80, 2.75	1.84	0.87, 3.90
Exposure to someone with resp. illness‡‡	None	1.83*	1.30, 2.58	1.73*	1.11, 2.67
Chronic GI condition	None	0.86	0.40, 1.87	1.45	0.66, 3.18
Chronic resp. condition	None	1.76*	1.09, 2.84	1.55	0.85, 2.82
Has diabetes	Does not	1.36	0.62, 2.97	0.85	0.26, 2.77
Took antibiotics in past 7 days	No	1.51	0.78, 2.92	1.36	0.58, 3.18
Prone to infection	None	0.73	0.26, 2.05	0.57	0.13, 2.39
Ate/drank , washed hands	Didn't eat/drink	1.07	0.64, 1.79	0.93	0.55, 1.58
Ate/drank, did not wash hands	Didn't eat/drink	1.08	0.71, 1.64	1.08	0.71, 1.65
Water recr., follow-up days 0-3	None	0.83	0.59, 1.16	0.89	0.60, 1.33
Canoeing	Motor boating			0.72	0.39, 1.33
Kayaking/rafting	Motor boating			0.58	0.32, 1.07
Rowing	Motor boating			0.76	0.35, 1.67
Fishing	Motor boating			1.24	0.65, 2.38
Swallowed ≥mouthful of water	Did not			10.89**	2.95, 40.20

* P <0.05 ** P<0.001

‡ ‡ Past 72 hours

Supplementary Material, Table 2c: results of multivariate logistic regression model of ear symptoms

Covariate	Reference /scale	All recreation groups AOR	All recreation groups 95% CI	Water recreation Groups only AOR	Water recreation Groups only 95% CI
CAWS	UNX	1.20	0.70, 2.07		
GUW	UNX	1.13	0.63, 2.01		
CAWS	GUW			1.03	0.65, 1.63
Age ≤10 yr	11-64 yrs	1.18	0.59, 2.36	1.14	0.50, 2.59
Age ≥65 yrs	11-64 yrs	0.19	0.03, 1.39	0.31	0.04, 2.29
Male	Female	0.69*	0.48, 0.99	0.66	0.43, 1.02
White	African Amer.	0.79	0.42, 1.49	0.63	0.27, 1.45
Hispanic	African Amer.	1.25	0.56, 2.78	0.67	0.22, 2.04
Other	African Amer.	0.47	0.18, 1.22	0.38	0.12, 1.24
Same water use: 5-10 days	0-4 days	1.59	0.96, 2.64	1.45	0.79, 2.68
Same water use: >10 days	0-4 days	1.26	0.74, 2.16	1.15	0.59, 2.24
Exposure to person w/ GI illness ‡‡	None	2.34*	1.22, 4.48	2.52*	1.12, 5.67
Exposure to person w/ resp. illness‡‡	None	0.87	0.55, 1.38	0.73	0.40, 1.34
Prone to infection	No	2.13	0.97, 4.67	0.91	0.22, 3.82
Diabetes	No	0.95	0.30, 3.08	0.47	0.06, 3.43
Perceived risk of CAWS recreation	0-10 scale	1.03	0.96, 1.10	1.05	0.97, 1.14
Ate/drank , washed hands	Didn't eat/drink	0.94	0.50, 1.76	0.83	0.44, 1.58
Ate/drank, did not wash hands	Didn't eat/drink	1.05	0.65, 1.71	1.09	0.66, 1.79
Water recreation, follow-up days 0-3	None	1.08	0.73, 1.58	1.11	0.70, 1.76
Canoeing	Motor boating		N/A	0.72	0.34, 1.54
Kayaking/rafting	Motor boating		N/A	0.60	0.28, 1.25
Rowing	Motor boating		N/A	0.96	0.41, 2.25
Fishing	Motor boating		N/A	1.56	0.70, 3.47
Face wetness score	0-4 scale		N/A	1.48*	1.20, 1.84

* P <0.05

‡ ‡ Past 72 hours

Supplementary Material, Table 2d: results of multivariate logistic regression model of eye symptoms

		3-group model		Water recreators	
**Covariate**		AOR	**95% CI**	**AOR**	**95% CI**
CAWS	UNX	1.50*	1.10, 2.06		
GUW	UNX	1.17	0.83, 1.65		
CAWS	GUW			1.34*	1.02, 1.77
Age ≤10 yr	11-64 yrs	0.22**	0.10, 0.49	0.20*	0.08, 0.56
Age ≥65 yrs	11-64 yrs	0.69	0.38, 1.25	0.85	0.41, 1.77
Male	Female	1.00	0.81, 1.24	1.00	0.78, 1.29
White	African Amer.	0.56*	0.40, 0.79	0.68	0.39, 1.19
Hispanic	African Amer.	1.06	0.68, 1.64	1.52	0.80, 2.88
Other	African Amer.	0.58*	0.37, 0.92	0.76	0.39, 1.48
Same water use: 5-10 days	0-4 days	1.03	0.73, 1.44	1.08	0.74, 1.58
Same water use: >10 days	0-4 days	0.77	0.54, 1.10	0.50*	0.30, 0.84
Exposure to person w/ GI illness‡‡	None	1.98*	1.29, 3.02	1.60	0.91, 2.80
Exposure to person w/ resp. illness‡‡	None	0.97	0.74, 1.28	0.89	0.63, 1.26
Exposure to person w/eye sympt..‡‡	None	1.11	0.50, 2.45	1.54	0.59, 3.97
Prone to infection	No	0.77	0.37, 1.58	0.71	0.29, 1.78
Diabetes	No	1.33	0.74, 2.38	0.89	0.38, 2.06
Perceived risk of CAWS recreation	0-10 scale	1.11**	1.06, 1.15	1.11**	1.05, 1.16
Ate/drank , washed hands	Didn't eat/drink	1.12	0.80, 1.58	1.01	0.71, 1.44
Ate/drank, did not wash hands	Didn't eat/drink	0.96	0.72, 1.28	0.89	0.67, 1.20
Water recreation, follow-up days 0-3	None	1.12	0.89, 1.41	1.20	0.92, 1.57
Canoeing	Motor boating		N/A	0.64*	0.42, 0.97
Kayaking/rafting	Motor boating		N/A	0.57*	0.38, 0.85
Rowing	Motor boating		N/A	0.55*	0.33, 0.92
Fishing	Motor boating		N/A	0.77	0.48, 1.25
Face wetness score	0-4 scale		N/A	1.12	0.97, 1.29
Hands wetness score	0-4 scale		N/A	1.21*	1.09, 1.35

* P <0.05 ** P<0.001

‡ ‡ Past 72 hours

Supplementary Material, Table 2e: results of multivariate logistic regression model of skin rash

		3-grp model		Water recreators	
**Covariate**	**Ref. /scale**	**AOR**	**95% CI**	**AOR**	**95% CI**
CAWS	UNX	0.86	0.64, 1.15		
GUW	UNX	0.72*	0.52, 1.00		
CAWS	GUW			1.18	0.91, 1.54
Age ≤10 yr	11-64 yrs	1.28	0.91, 1.81	1.22	0.78, 1.91
Age ≥65 yrs	11-64 yrs	0.52	0.26, 1.02	0.54	0.22, ,1.34
Male	Female	0.86	0.71, 1.05	0.87	0.68 1.10
White	African Amer.	0.63*	0.45, 0.88	0.59	0.34, 1.01
Hispanic	African Amer.	0.77	0.48. 1.22	0.88	0.46, 1.70
Other	African Amer.	1.21	0.82, 1.79	1.10	0.60, 2.01
Same water use: 5-10 days	0-4 days	0.76	0.54, 1.09	0.79	0.52, 1.21
Same water use: >10 days	0-4 days	0.99	0.73, 1.35	0.74	0.48, 1.14
Contact with dog or cat‡	None	0.93	0.76, 1.15	0.98	0.76, 1.26
Antibiotic use, past 7 days	None	1.36	0.89, 2.08	1.33	0.78, 2.26
Sunburn at time of enrollment	None	1.73**	1.31, 2.27	1.71*	1.26, 2.33
Cuts at the time of enrollment	None	1.33*	1.06, 1.67	1.31	1.00, 1.72
Bug bites at time of enrollment	None	2.25**	1.82, 2.78	2.20**	1.71, 2.83
Ate shellfish/sushi ‡	None	0.90	0.60, 1.36	1.03	0.63, 1.69
Prone to infection	No	1.86*	1.16, 2.98	1.64	0.86, 3.11
Diabetic	No	1.17	0.65, 2.10	1.18	0.56, 2.48
Perceived risk of CAWS recreation	0-10 scale	1.03	0.99, 1.07	1.02	0.97, 1.07
Ate/drank , washed hands	Didn't eat/drink	0.82	0.57, 1.19	0.79	0.54, 1.16
Ate/drank, did not wash hands	Didn't eat/drink	1.13	0.86, 1.49	1.14	0.86, 1.51
Water recreation, follow-up days 0-3	None	1.34*	1.06, 1.69	1.38*	1.03, 1.84
Canoeing	Motor boating		N/A	0.75	0.48, 1.16
Kayaking/rafting	Motor boating		N/A	0.80	0.52, 1.23
Rowing	Motor boating		N/A	0.98	0.60, 1.61
Fishing	Motor boating		N/A	1.00	0.62, 1.61
Wetness score	0-16 scale		N/A	1.02	0.98, 1.06

* P <0.05 ** P<0.001

‡ Past 48 hours prior to enrollment

Supplementary Material, Table 3: alternative definitions of exposure in analyses of 2-group models of AGI

Variable for water exposure	AGI and exposure OR (95% CI)	AGI and group OR (95% CI)
**Face/head wetness**		
None vs. any	1.59 (1.23, 2.05)	0.97 (0.74, 1.22)
None, drop vs. splash, drench, submerge	1.85 (1.38, 2.47)	1.01 (0.79, 1.30)
None, drop, splash vs. drench, submerge	2.25 (1.28, 3.95)	1.05 (0.82, 1.35)
None, drop, splash, drench vs. submerge	2.41 (1.25, 4.69)	1.05 (0.82, 1.35)
**Swallow water**		
None vs. any	1.57 (0.94, 2.63)	1.01 (0.78, 1.29)
None, drop vs. teaspoon, mouthful	2.79 (1.41, 5.53)	1.02 (0.79, 1.31)
None, drop, teaspoon vs. mouthful	5.74 (2.05, 16.0)	1.02 (0.80, 1.31)

All associations were obtained from the multivariate model presented in Supplementary Material Table 2a. The seven sets of results presented here were from separate models, each using a different exposure term.

<u>Supplementary Material Figures 1a and 1b: CHEERS recruiting locations</u>

Recruiting locations on the CAWS and nearby GUW locations are presented in the following two figures (CHEERS North Side and CHEERS South Side). Additional GUW recruiting locations were the Fox River, Des Plaines River, Du Page River, as well as at the following inland lakes: Busse Woods, Crystal Lake, Lake Arlington, Maple Lake, Mastodon Lake, the Skokie Lagoons and Tampier Lake.

CHEERS North Side

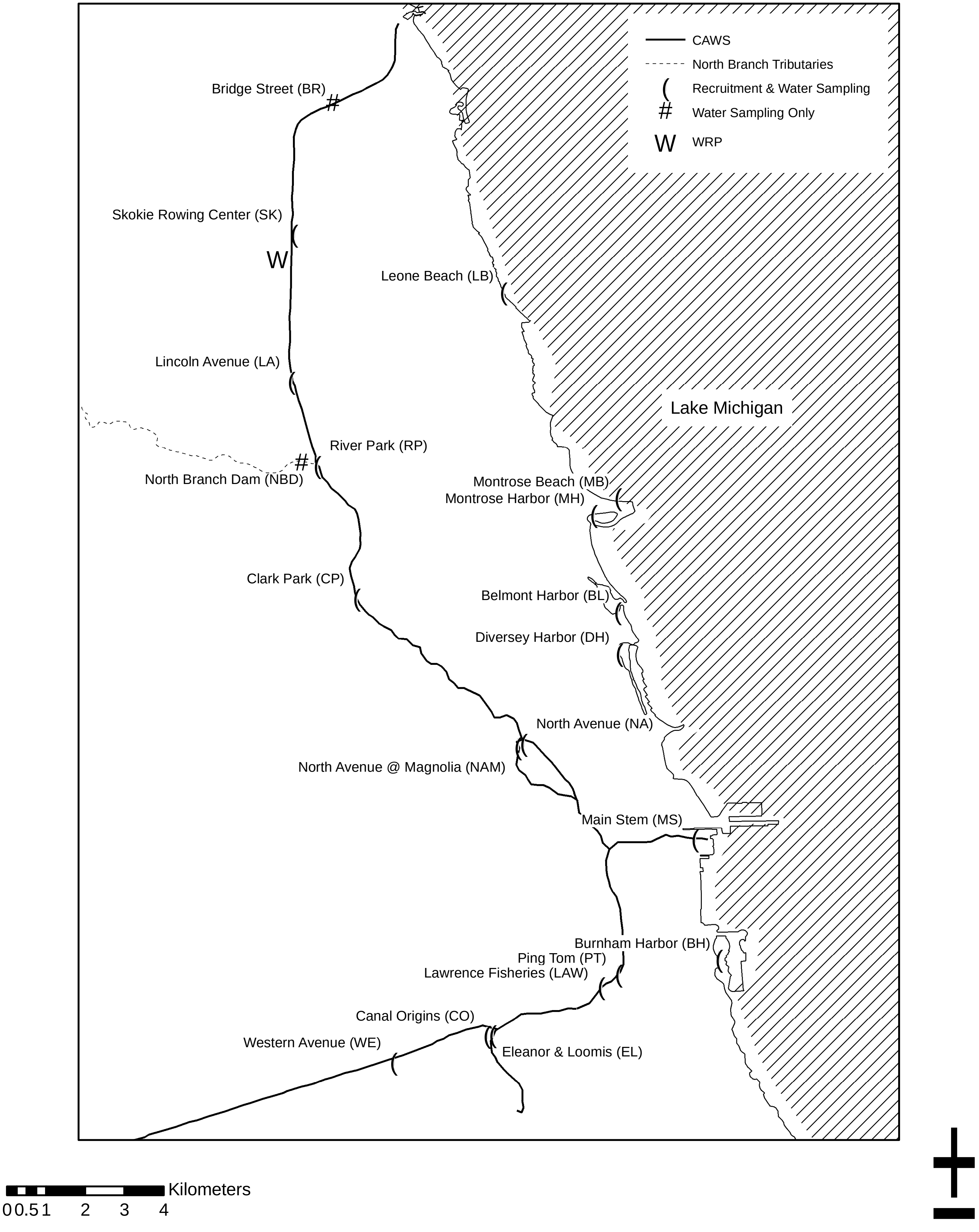

CHEERS South Side

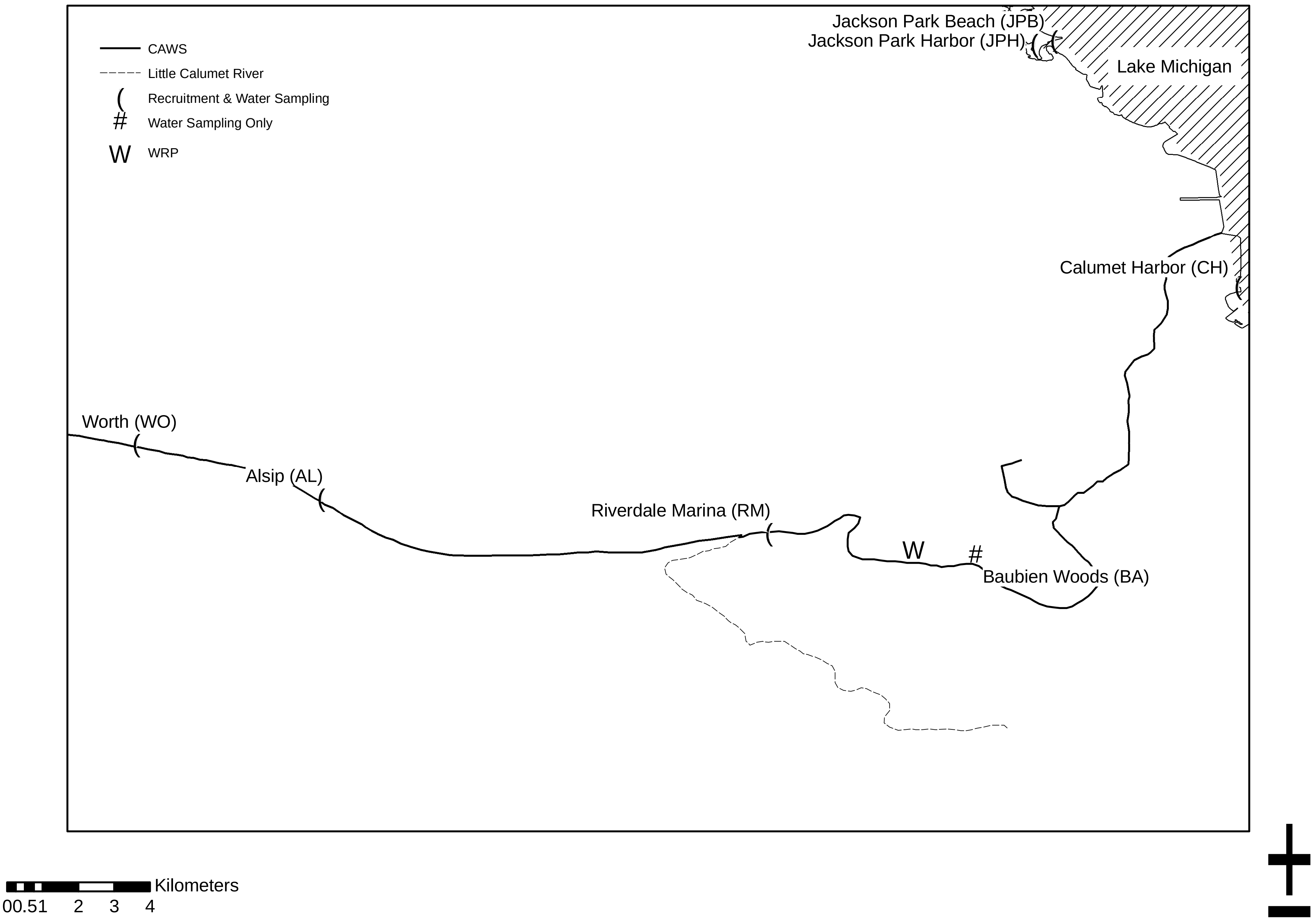

Supplementary Material Figure 2: Participant Enrolment and Attrition

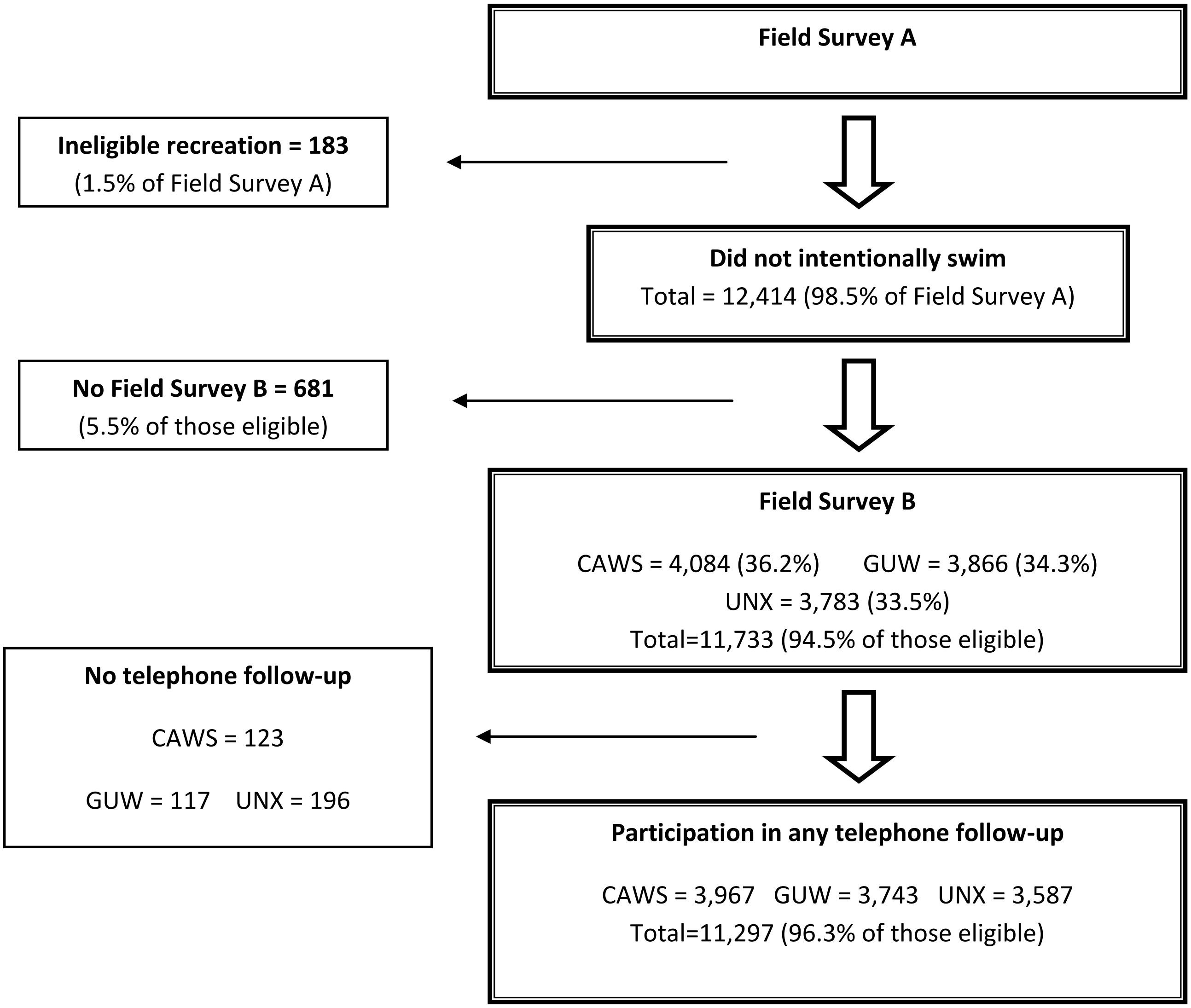

Supplementary material Figure 3a: Survival curve, acute respiratory illness (ARI)

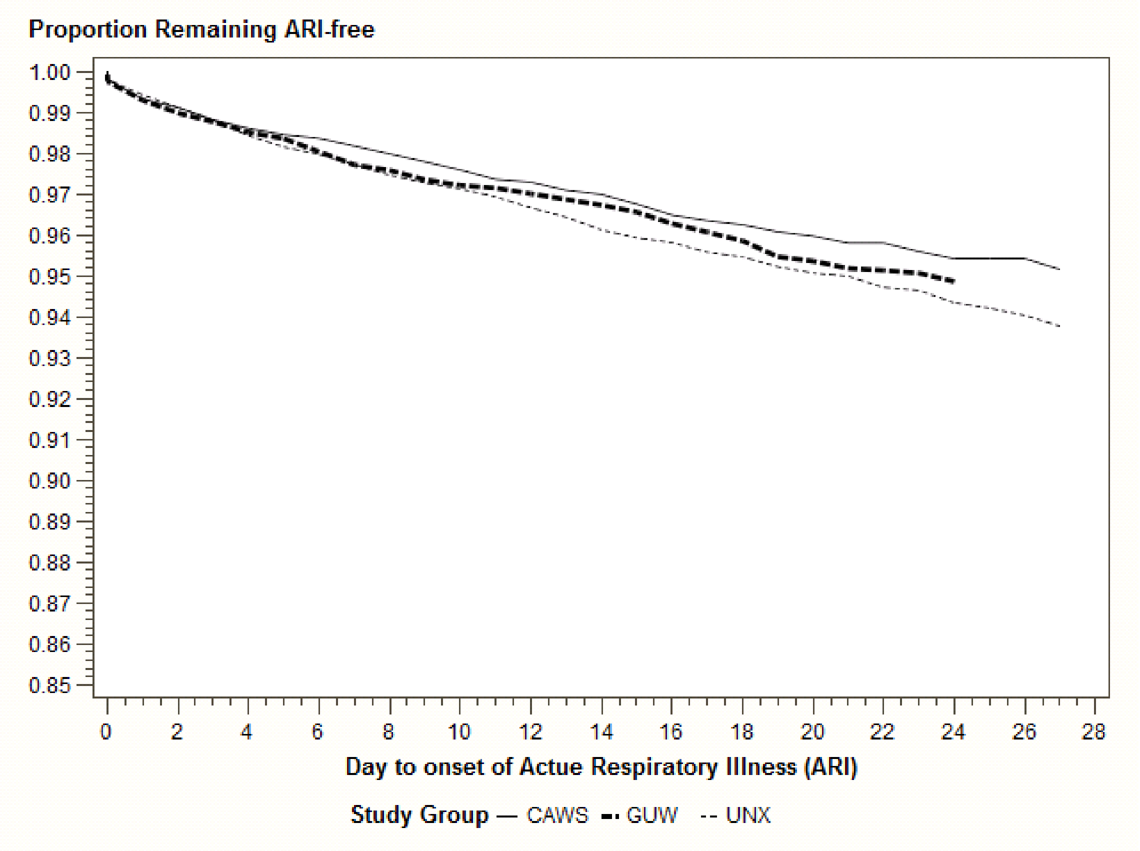

Supplementary material Figure 3b: Survival curve, ear symptoms

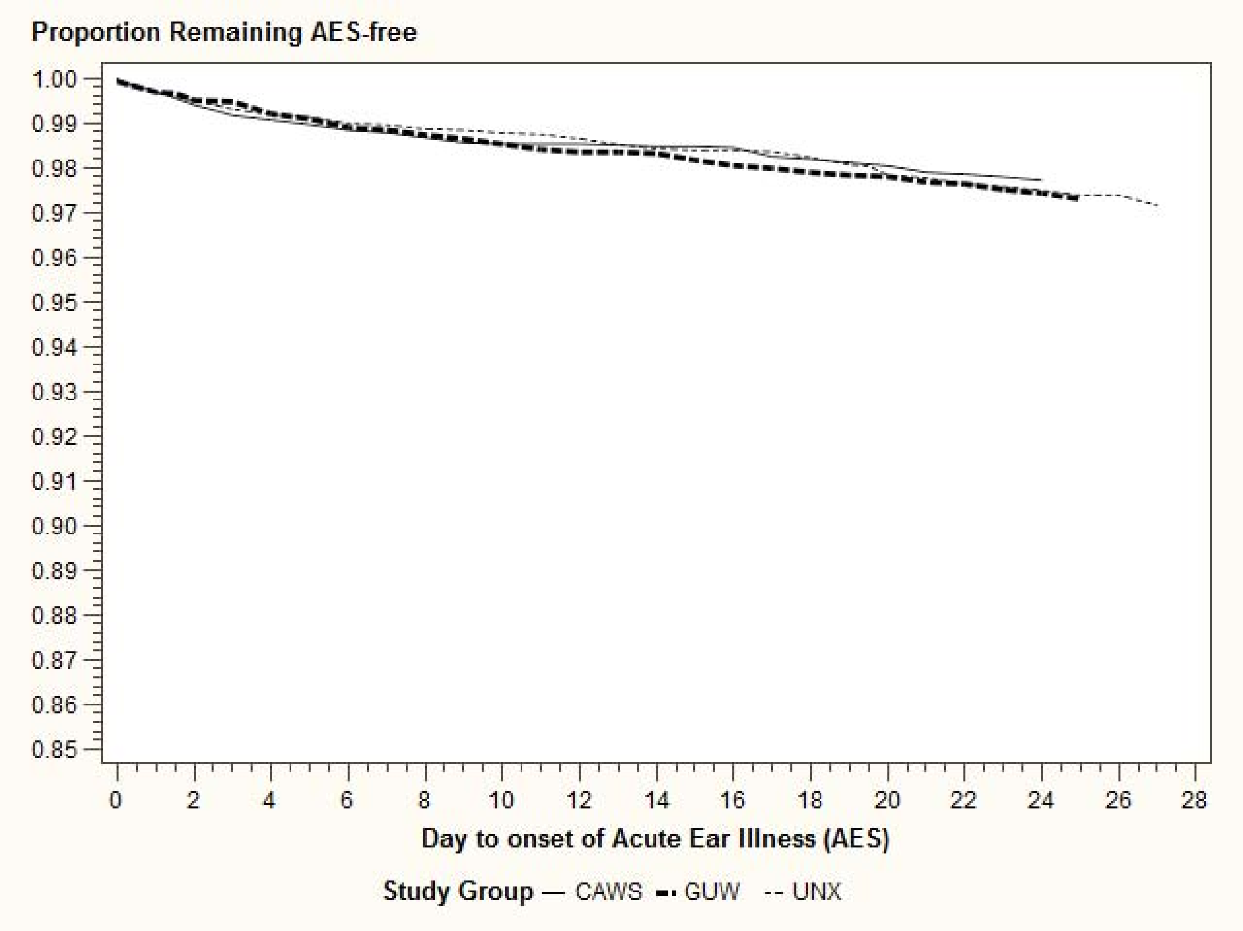

Supplementary material Figure 3c: Survival curve, eye symptoms

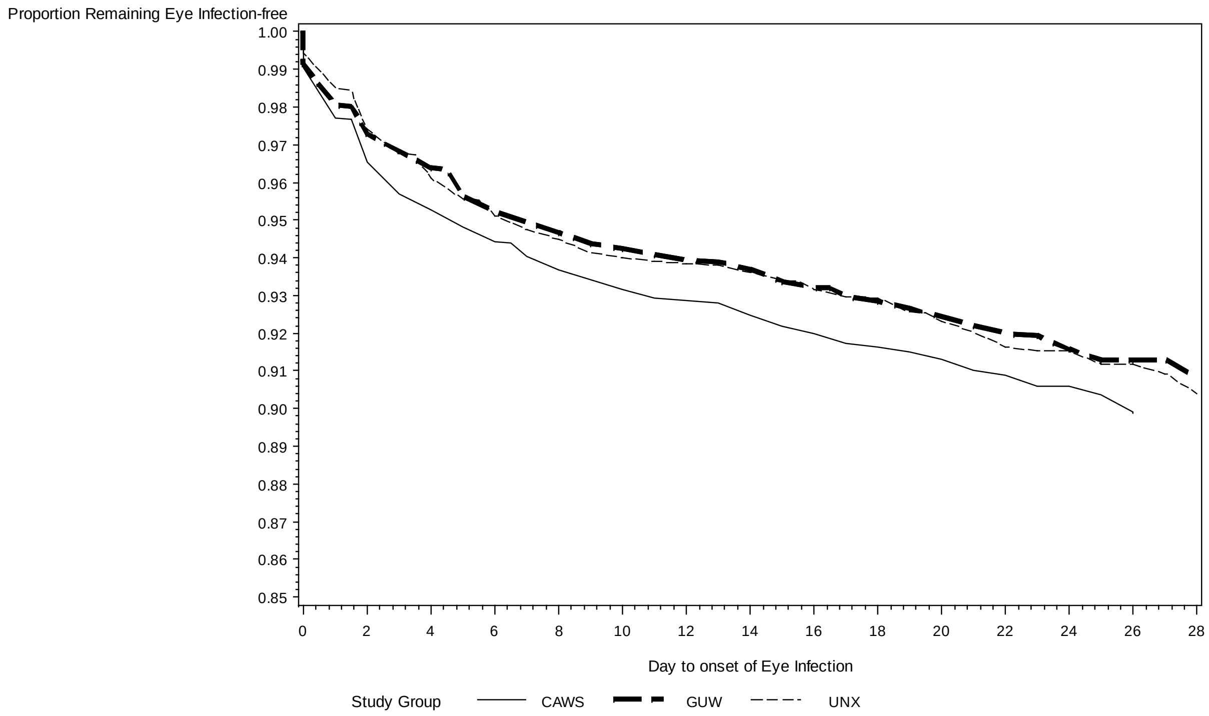

Days to onset of eye symptoms

Supplementary material Figure 3d: Survival curve, skin rash

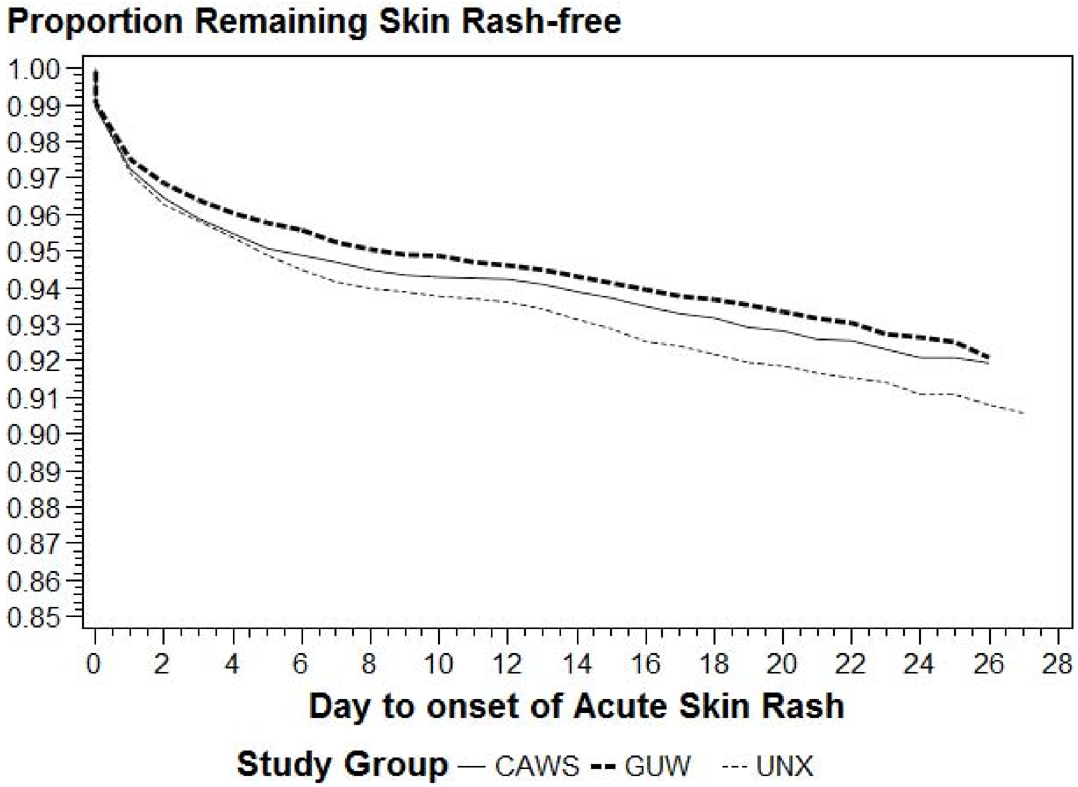

Supplement: (410 KB) PDF [file ehp.1103934.s001.pdf]
